# The Core Eudicot Boom Registered in Myanmar Amber

**DOI:** 10.1038/s41598-018-35100-4

**Published:** 2018-11-13

**Authors:** Zhong-Jian Liu, Diying Huang, Chenyang Cai, Xin Wang

**Affiliations:** 1 Key Laboratory of National Forestry and Grassland Administration for Orchid Conservation and Utilization at College of Landscape Architecture, Fujian Agriculture and Forestry University, Fuzhou, 350002 China; 2 State Key Laboratory of Palaeobiology and Stratigraphy, Nanjing Institute of Geology and Palaeontology and Center for Excellence in Life and Paleoenvironment, Chinese Academy of Sciences, Nanjing, 210008 China; 3 CAS Key Laboratory of Economic Stratigraphy and Paleogeography, Nanjing Institute of Geology and Palaeontology and Center for Excellence in Life and Paleoenvironment, Chinese Academy of Sciences, Nanjing, 210008 China

## Abstract

A perfect flower in a mid-Cretaceous (early Cenomanian) Myanmar amber is described as *Lijinganthus revoluta* gen. et sp. nov. The fossil flower is actinomorphic and pentamerous, including calyx, corolla, stamens, and gynoecium. The sepals are tiny, while the petals are large and revolute. The stamens are dorsifixed, filamentous, and each has a longitudinally dehiscing bisporangiate anther. The gynoecium is in the centre of the flower, composed of three fused carpels with a stout style. *Lijinganthus revoluta* gen. et sp. nov. demonstrates a great resemblance to the flowers of Pentapetalae (Eudicots), adding new information to the enigmatic early evolutionary history of Pentapetalae and Eudicots.

## Introduction

**Figure 1:**
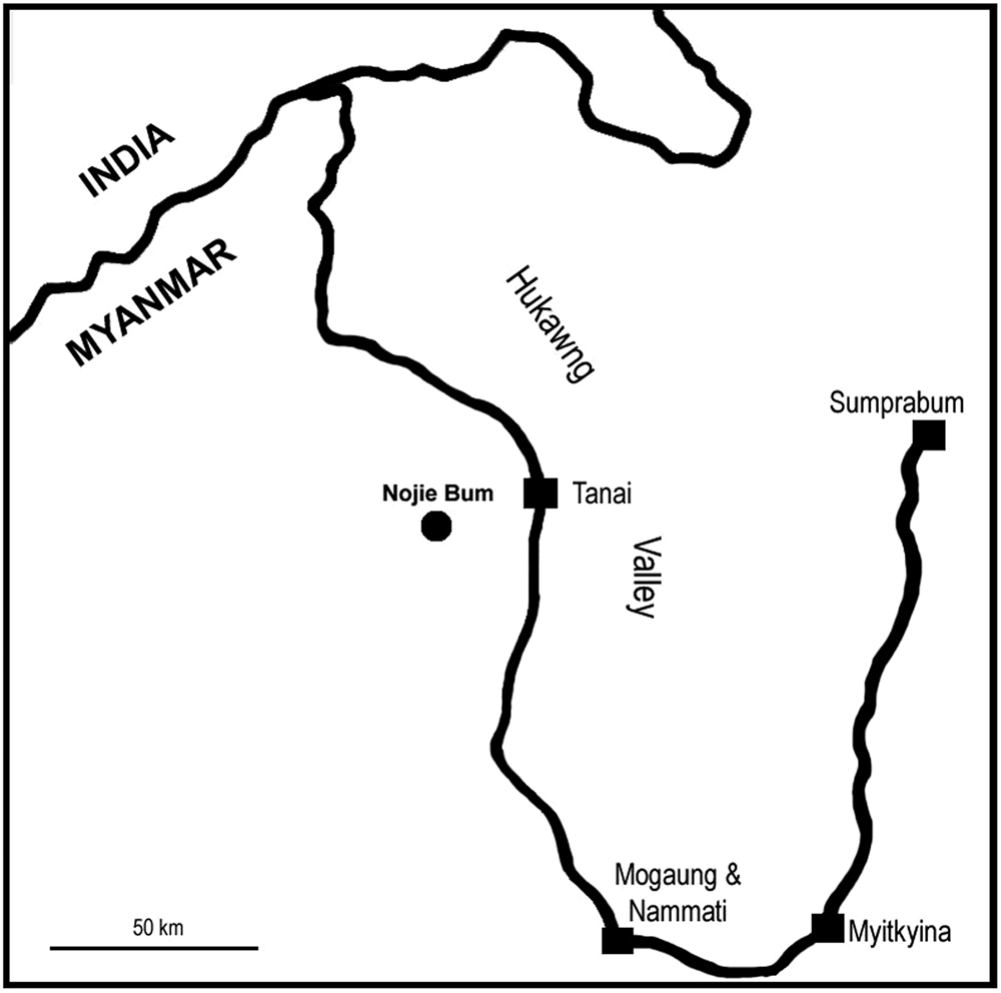
Nojie Bum, the fossil locality (round dot) of *Lijinganthus revoluta* gen. et sp. nov., in northern Myanmar. The major cities are shown as squares.

An increasing number of insects and plants have been reported in a mid-Cretaceous Myanmar ambers^1–23^. Among them, fossil flowers have shed light on the diversification of angiosperms during this important radiating period for angiosperms^2,18–21,24^. Core Eudicots comprise a major portion of the species diversity in extant angiosperms, and they underwent a rapid increase in diversity and abundance at the transition between the Early and Late Cretaceous^19,25–28^. Hitherto, the earliest record of a flower with distinct sepals and petals is approximately 94 Ma (the Cenomanian)^29,30^ (However, according to the latest study (Manchester *et al*.^27^), some fruit specimens belonging to the same taxon may be dated back to 105 Ma (the Albian)). Various molecular clocks indicate that angiosperms and Eudicots have a significantly earlier origin than the earliest fossil record indicates^31–33^. The gap between these estimations and the fossil record makes many conclusions in angiosperm systematics tentative. Here, we describe a new flower, *Lijinganthus revoluta* gen. et sp. nov., from an earliest Cenomanian-latest Albian (98.79 Ma)^34^ amber, which was collected from Noije Bum 2001 Summit Site, Hukawng Valley, Kachin, Myanmar (26°20′N, 96°36′E) (Fig. 1). This bisexual, pentamerous, actinomorphic flower with distinct sepals and petals, filamentous bisporangiate stamens, and trimerous gynoecium with superior ovary and axile placentation is unique, demonstrating a great resemblance to Pentapetalae (Core Eudicots) and thus shedding a new light on the evolution of Core Eudicots. *Lijinganthus*, as a representative of Pentapetalae, is among the first Core Eudicots. Together with contemporary fossil finds^19,25–28^, *Lijinganthus* suggests a Core Eudicot Boom at the transition from the Early to Late Cretaceous and helps to narrow the gap between fossil record and molecular clock estimates. The presence of a nectary disk in *Lijinganthus* suggests the possibility of insect-mediated pollination.

## Results

**Systematic palaeontology**

**Angiospermae**

**Eudicots**

**Pentapetalae**

**Family** ***incertae sedis***

***Lijinganthus*** gen. nov.

**Type species**: *Lijinganthus revoluta* gen. et sp. nov.

**Diagnosis**: Flower actinomorphic, bisexual, with one whorl of five sepals, one whorl of five petals, androecium, and gynoecium. The calyx consisting of five distinct, small sepals with entire margins and rounded apices. The corolla consisting of five distinct, large, elongated oval, entire-margined revolute petals with rounded apices, alternating with the sepals. Androecium diplostemonous, filamentous. The anthers bisporangiate, introrse, dorsifixed, dehiscing by longitudinal slits. Nectary disk at the base of the gynoecium. The gynoecium tricarpous, lacking an obvious style and probably with axile placentation.

**Figure 2:**
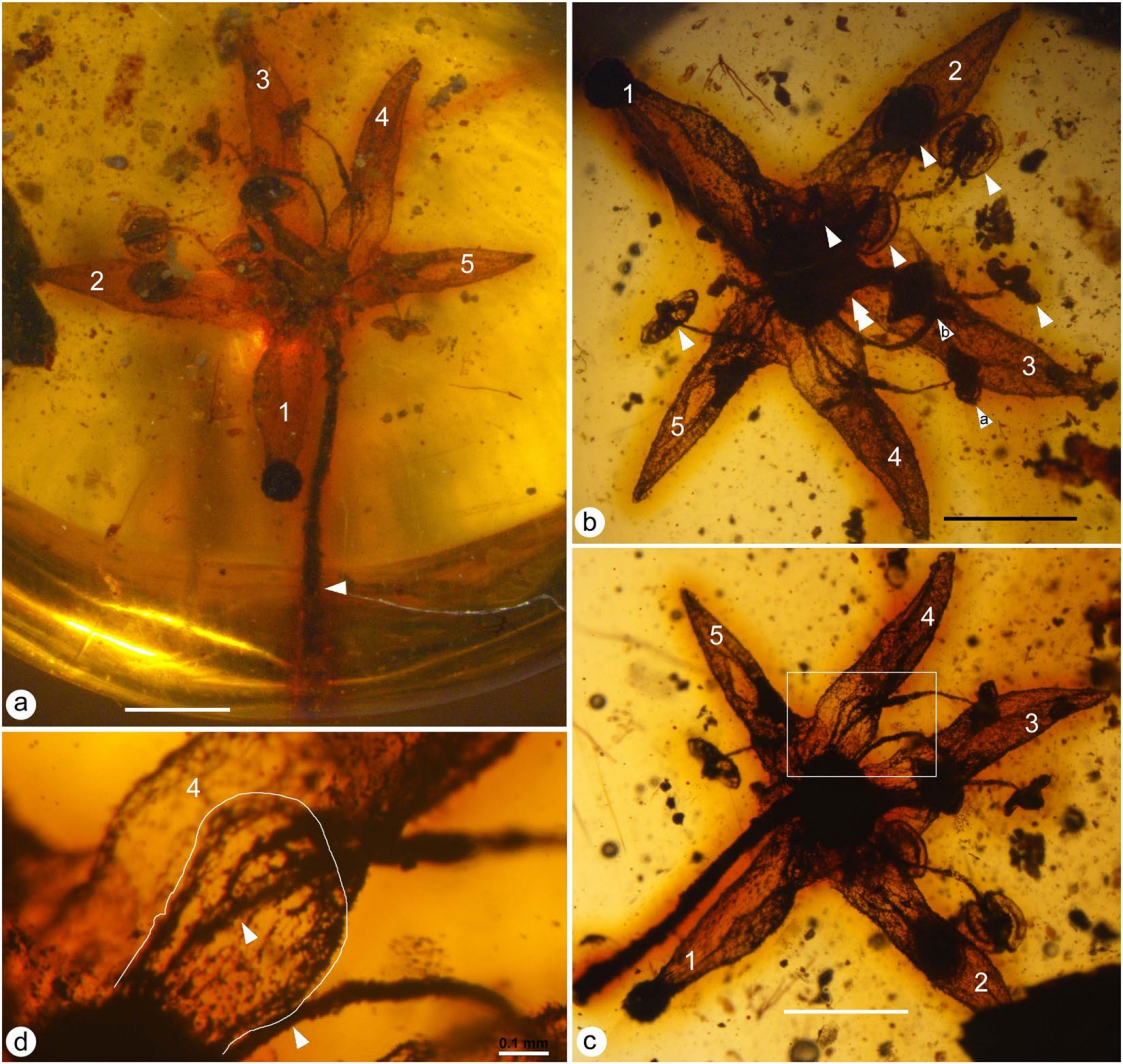
*Lijinganthus revoluta* gen. et sp. nov. embedded in a Myanmar amber. (**a**) Side view of the flower, showing physically connected pedicel (white triangle), petals (1–5), and stamens. Scale bar = 1 mm. (**b**) Top view of the flower, showing petals (1–5), anthers (white triangles), and ovary (double triangle). Note the relationship between stamens (**a**,**b**) and Petal 4. Scale bar = 1 mm. (**c**) Bottom view of the flower, showing revolute petals (1–5) and stamens. Scale bar = 1 mm. (**d**) Detailed view of the rectangular region in 1c, showing the relationship among sepal (white line), petal (4), and 2 filaments (white triangles). Scale bar = 0.1 mm.

**Figure 3:**
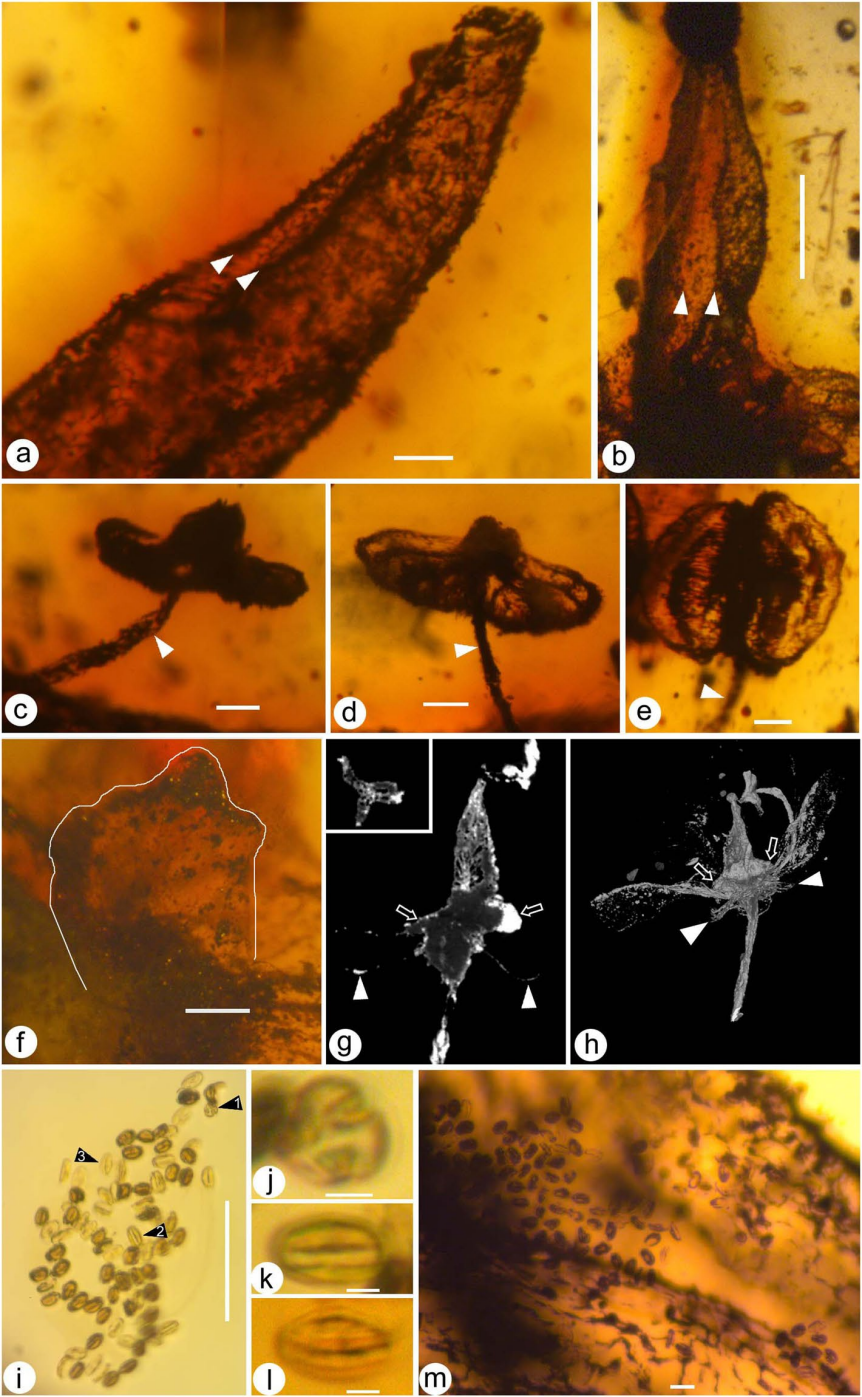
Detailed view of *Lijinganthus revoluta* gen. et sp. nov. embedded in a Myanmar amber. (**a**) Abaxial view of the distal portion of Petal 4, showing the revolute form and the margins (white triangles) of the petal. Scale bar = 0.1 mm. (**b**) Abaxial view of the distal portion of Petal 1, showing the margins (white triangles) of revolute petal. Scale bar = 0.5 mm. (**c**–**e**) Top (**c**,**d**) and adaxial (**e**) views of the dorsifixed bisporangiate anthers on the termini of slender filaments (white triangles). Scale bar = 0.1 mm. (**f**) Detailed view of a sepal (white line). Scale bar = 0.1 mm. (**g**) Micro-CT virtual section of the gynoecium, showing the conical profile of the ovary, nectary disk (arrows), inconspicuous style, and petals (white triangles). The inset shows the cross section of the ovary near the tip. (**h**) Micro-CT virtual view showing relationship among the pedicel, sepals (white triangles), petals, stamen, nectary disk (arrows), conical ovary, and inconspicuous style in the centre. (**i**) Approximately 70 tricolpate pollen grains closely associated with the flower. Scale bar = 0.1 mm. (**j**–**l**) Different views of tricolpate pollen grains, enlarged from those marked as 1–3, respectively, in (**i**). (**j**) polar view; (**k**,**l**) equatorial views. Scale bar = 5 μm. (**m**) Numerous pollen grains adjacent to one of the petals of the flower. Scale bar = 20 μm.

**Figure 4:**
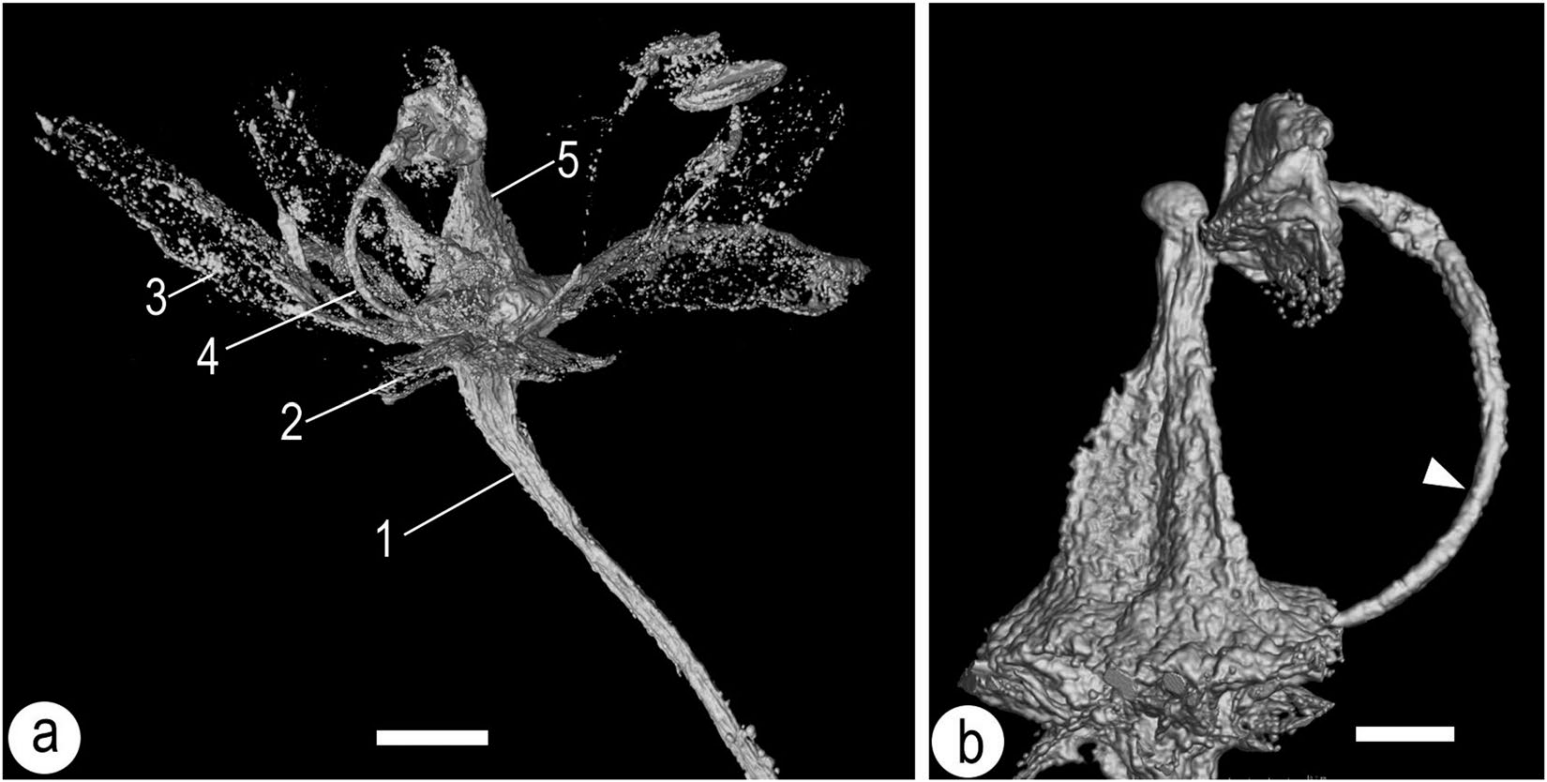
Detailed view of *Lijinganthus revoluta* gen. et sp. nov. embedded in a Myanmar amber. (**a**) Micro-CT visualization of the flower. 1, pedicel; 2, sepal; 3, petal; 4, stamen; 5, ovary. Scale bar = 0.5 mm. (**b**) Detailed view of a stamen and the ovary, showing the slender filament (white triangles) attached to the dorsal of the anther that opens along two adaxial longitudinal slits. Scale bar = 0.2 mm.

**Figure 5:**
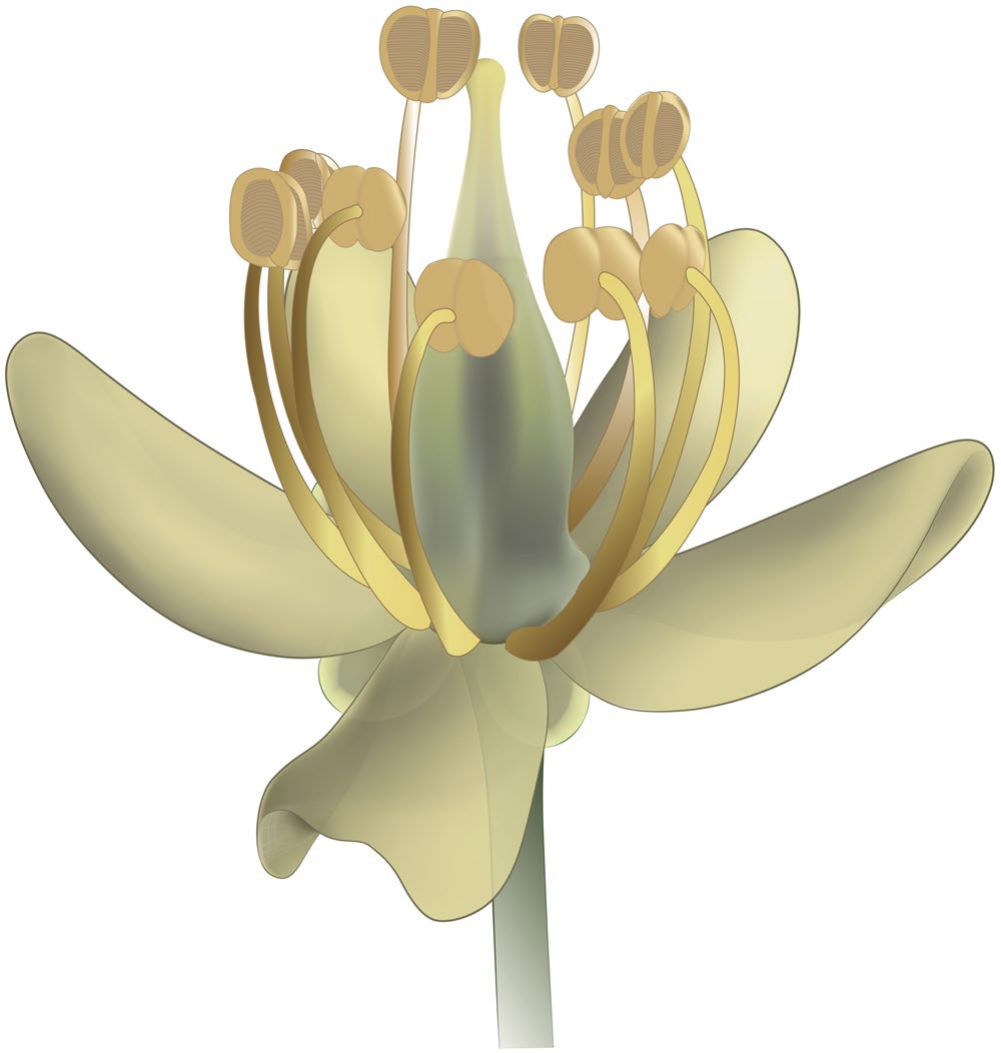
Reconstruction of *Lijinganthus revoluta* gen. et sp. nov.

***Lijinganthus revoluta*** gen. et sp. nov.

(Figures 2, 3, 4, 5)

**Description:** The flower (including the pedicel) is 6.5 mm long and 4.8 mm wide. The flower includes a pedicel, sepals, petals, stamens, and a gynoecium (Figs 2a–c, 3h and 4a). The pedicle is 0.2 mm in diameter and 3.8 mm long, slightly tapering distally. There are five distinct sepals, each 0.4-0.5 mm long and 0.32-0.33 mm wide, with an obtuse or round tip (Figs 2d, 3f,h and 4a). Alternating with the sepals are five distinct petals, each approximately 1.8 mm long and 0.67 mm wide, revolute (Figs 2a–d, 3a,b and 4a). At least eight distinct stamens are seen inserted on the nectary disk at the base of the ovary, each including a slender filament and a dorsifixed bisporangiate anther (Figs 2a–d, 3c–e,h and 4a,b). The filament is approximately 43 μm wide, 1.3 mm long, tapering distally (Figs 2a–d, 3c–e and 4a,b). The anther dehisces longitudinally, 0.5 mm wide and 0.5 mm long, without *in situ* pollen grains (Figs 3c–e,h and 4b). Several clumps of a single type of pollen grains are present in the same amber block and closely associated with the flower (Figs 3i,m and S1a–q). Pollen grains are tricolpate, 14–21 μm long and 10–12 μm in diameter (Fig. 3i–l). Nectary surrounds the base of the ovary (Fig. 3g–h). The gynoecium is situated centrally, including an ovary that tapers distally with no obvious style (Figs 3g,h and 4a,b). The ovary appears to be composed of three fused carpels, probably with axile placentation (Fig. 3g). The ovary is approximately 0.77 mm in diameter, with its tip only 0.13 mm in diameter, and round triangular in cross view (Figs 2b,c, 3g,h, 4b and 5).

**Etymology**: The generic name, *Lijinganthus*, is dedicated to poetess Ms. Jing Li (1967–2015) for her talent and poetry, and “*anthus*” from the Latin “anthos” (flower). The specific epithet *revoluta* is for the revolute form of the petals.

**Type specie**s: *Lijinganthus revoluta* gen. et sp. nov.

**Holotype:** PB22841.

**Deposition**: The Nanjing Institute of Geology and Palaeontology, Chines Academy of Sciences.

**Type locality**: Noije Bum 2001 Summit Site, Hukawng, Kachin, Myanmar (26°21′33.41″N, 96°43′11.88″E).

**Horizon**: the earliest Cenomanian-latest Albian, Cretaceous (98.79 Ma).

**Remarks**: Although only eight stamens are preserved in the flower, the spatial relationship between stamens and petals (Fig. 2d) and the presence of five petals (Fig. 2a–c) suggest that the total number of original stamens in *Lijinganthus* should be ten. Further research need to be done to confirm this.

The character combination of the fossil does not allow us to assign it to any known fossil or extant genus of angiosperms, thereby justifying a new genus.

## Discussion

### Comparison with extant angiosperms

The occurrence of over 800 single type of tricolpate pollen grains closely associated with *Lijinganthus revoluta* gen. et sp. nov. (preserved in the same block, and their distances from the flower range from 2.5 to 0 mm (Figs 3i,m and S1a–q)) strongly suggests a eudicot affinity for *Lijinganthus*, as tricolpate pollen grains are a characteristic feature of Eudicots, which are frequently termed Tricolpates. Among Eudicots, the occurrence of distinct calyx and corolla in *Lijinganthus* distinguishes it from the basal eudicots (with undifferentiated perianth)^30^ and Gunnerales (lacking perianth)^35^, suggesting that *Lijinganthus* belongs to the Pentapetalae in Core Eudicots. Several features of *Lijinganthus* are also seen in Crassulaceae (Saxifragales), but the latter has a basifixed anther, >3 more or less free carpels, decurrent stigma, and parietal placentation^36,37^ and is thus distinct from *Lijinganthus* with dorsifixed anther, fused carpels, capitate stigma, and axile placentation. Although the presence of distinct sepals and petals suggest that *Lijinganthus* is most likely related to the Superrosids, and its tricarpous gynoecium suggests a possible affinity to Malpighiales, we think it is premature to assign *Lijinganthus* to any group within Pentapetalae, at least for the time being.

### Comparison with the Early Flowers of Core Eudicots

Previously, the earliest record of a flower with distinct calyx and corolla was marked by a fossil flower named “Rose Creek flower” from the Albian-Cenomanian^30^, which was recognized by Basinger and Dilcher in 1984^29^ and reworked on and renamed as *Dakotanthus cordiformis* by Manhcester *et al*. (2018) with additional specimens (especially of fruits) using CT technology^27^. *Lijinganthus* is similar to *Dakotanthus cordiformis* in distinct pentamerous symmetry (5 sepals and 5 petals) and bisexuality, but it differs from the latter in long (rather than ovate) petals, a slender (rather than stout) filament, a bisporangiate (rather than tetrasporangiate) anther, tricolpate (rather than tricolporate) pollen grains, a trimerous (rather than pentamerous) gynoecium, and 1 (rather than 5) style^27,29^. *Dakotanthus cordiformis* is initially interpreted as approximately 94 Ma old^29^, but recent study, with more specimens (especially of fruits) from strata other than the original locality (Rose Creek), suggests that the age of *Dakotanthus cordiformis* may be extended to 105 Ma^27^. *Dakotanthus cordiformis* was considered to be “the first fossils with unequivocal features of core eudicots” from the Albian-Cenomanian^30^. The age of 98.79 Ma of *Lijinganthus* places our flower near the Albian/Cenomanian boundary (97.2 Ma to100.5 Ma by various authors). The concentrated occurrence of various flowers of different lineages (*Lachnociona terriae* (Brunelliaceae/Cunoniaceae (Oxalidaels) + Rosids + Saxifragles), *Tropidogyne pikei* and *T*. *pentaptera* (Cunoniaceae), *Eoëpigynia burmensis* (Cornaceae), *Dakotanthus cordiformis* (Quillajaceae)) and *Lijinganthus* at about the same time^19,25–28^ seems to suggest the Core Eudicots underwent a rapid diversification (“Core Eudicot Boom”) at the very beginning of the Late Cretaceous (Table 1). It is intriguing to investigate whether there is a coupling between this important plant event and the Upper Albian OAE 1d event (including rapid CO_2_ concentration rising)^38^ as well as the decline of Gnetales and Bennettitales.

**Table 1:** Comparison among the flowers of pioneer Core Eudicots in the mid-Cretaceous (Albian-Cenomanian).

	Symmetry	Gender	Sepal	Petal	Stamen	Filament	Pollen sac	Pollen grain	Floral cup	Ovary	Placentation	Carpel	Style	Preservation media	Locality	Reference
*Lijinganthus revoluta*	actinomorphic	bisexual	5, free	5, free, revolute	8(10?), dorsifixed, introse	slender	2	tricolpate	None	superior	axile	3, fused		amber	Burma	This study
*Dakotanthus cordiformis*	actinomorphic	bisexual	5, free	5, free, spatulate	10?, dorsifixed	stout	4	tricolporate		superior	axile	5, fused		siltstone	USA	Manchester *et al*.^27^
*Lachnociona terriae*	actinomorphic	unisexual	5, free	none	10	slender			present	superi, half-inferior		5, fused or not	connivent	Amber	Burma	Poinar *et al*.^19^
*Tropidogyne pentaptera*	actinomorphic	bisexual	5, slightly fused	0	5? dorsifixed	short, slender			present	inferior		2, fused	2	amber	Burma	Poinar *et al*.^28^
*Eoȅpigynia burmensis*	actinomorphic	bisexual	4, fused	4, free, valvate	4, dorsifixed, introse	slender	?	tricolporate?	present	inferior	?	fused		amber	Burma	Poinar *et al*.^18^
*Caliciflora mauldinensis*	actinomorphic	Bisexual	5, free, revolute-valvate	5, free, keeled-conduplic-ate	8, dorsifixed	short or none	4	tricolporate	present	superior	marginal?	3, free		siltstone	USA	Friis *et al*.^26^

The presence of nectary disk in *Lijinganthus* suggests that insects may have begun interacting with flowers by the Cenomanian. Whether such an interaction is a major driving force for the diversification of Core Eudicots is apparently a question deserving further investigation.

### Earlier Origins

Various molecular clock studies have indicated that Eudicots and angiosperms originated much earlier than formerly assumed^31–33^. The discovery of *Lijinganthus* from the late Albian-Early Cenomanian (98.79 Ma) adds to the diversity and abundance of early Core Eudicots and helps to reconcile the conflicts between different schools and studies. Compatible with the molecular studies, the early age of *Lijinganthus*, together with recently found earlier-than-recorded fossils of Poaceae^39^ and Solanaceae^40^ as well as various contemporaneous Core Eudicots^19,25–28^ points to a cryptic and unexpectedly longer history of angiosperms and Eudicots. It is noteworthy that this conclusion is compatible with previously documented pre-Cretaceous traces of angiosperms^41–48^.

## Conclusions

*Lijinganthus revoluta* gen. et sp. nov. and other contemporaneous fossil flowers suggest a Core Eudicot Boom at the very beginning of the Late Cretaceous. Increasing number of reports of early fossil flowers seem to converge earlier origins of various lineages that have been predicted by molecular clocks.

## Methods

The specimen was collected from Noije Bum 2001 Summit Site, Hukawng Valley, Kachin, Myanmar (26°20′N, 96°36′E) (Fig. 1). Paleontological studies indicate that the specimen belongs to the earliest Cenomanian-latest Albian, Early Cretaceous (98.79 Ma)^34^, which is generally agreed on by Poinar *et al*.^28^ and Xing *et al*.^49^. Two parallel planes were made on the amber sample before observations. Observations and photographs were made with a Nikon SMZ1500 stereoscopic microscope at the Nanjing Institute of Geology and Palaeontology, Nanjing, China. Micro-CT was performed using a Zeiss Xradia 520 versa X-ray microscope at the Nanjing Institute of Geology and Palaeontology, Nanjing, China. The 3D reconstruction and virtual sections were generated using VGStudio MAX 3.0. All figures were organized for publication using Photoshop 7.0.

## Electronic supplementary material

Supplementary Information

## Data Availability

The holotype (PB22841) is accessible in the palaeobotanical collection of the Nanjing Institute of Geology and Palaeontology, Chines Academy of Sciences, 39 Beijing Dong Road, Nanjing 210008, China.
