# Supplementary material for: The Core Eudicot Boom Registered in Myanmar Amber
Source: Sci Rep. 2018 Nov 13;8:16765. doi: 10.1038/s41598-018-35100-4 (PMC6233203; doi:10.1038/s41598-018-35100-4)
Supplement: Supplementary file 1 — Supplementary Information [file 41598_2018_35100_MOESM1_ESM.pdf]

Supplementary Information

The Core Eudicot Boom Registered in Myanmar Amber

Zhong-Jian Liu[1,], Diying Huang[2], Chenyang Cai[2], Xin Wang[3,*]

[1]Key Laboratory of National Forestry and Grassland Administration for Orchid Conservation and Utilization at College of Landscape Architecture, Fujian Agriculture and Forestry University, Fuzhou 350002, China

[2]State Key Laboratory of Palaeobiology and Stratigraphy, Nanjing Institute of Geology and Palaeontology and Center for Excellence in Life and Paleoenvironment, Chinese Academy of Sciences, Nanjing 210008, China

[3]CAS Key Laboratory of Economic Stratigraphy and Paleogeography, Nanjing Institute of Geology and Palaeontology and Center for Excellence in Life and Paleoenvironment, Chinese Academy of Sciences, Nanjing 210008, China

[*]Email: xinwang@nigpas.ac.cn, ORCID: 0000-0002-4053-5515

One of the unique features of Lijinganthus revoluta gen. et sp. nov. is the associated pollen grain clumps. There are at least 22 clumps of pollen grains associated with this flower (Fig. S1a). The pollen grains are in clumps (Figs. S1a-q). The total number of all associated pollen grains are over 800, and they all belong to the same tricolpate type.

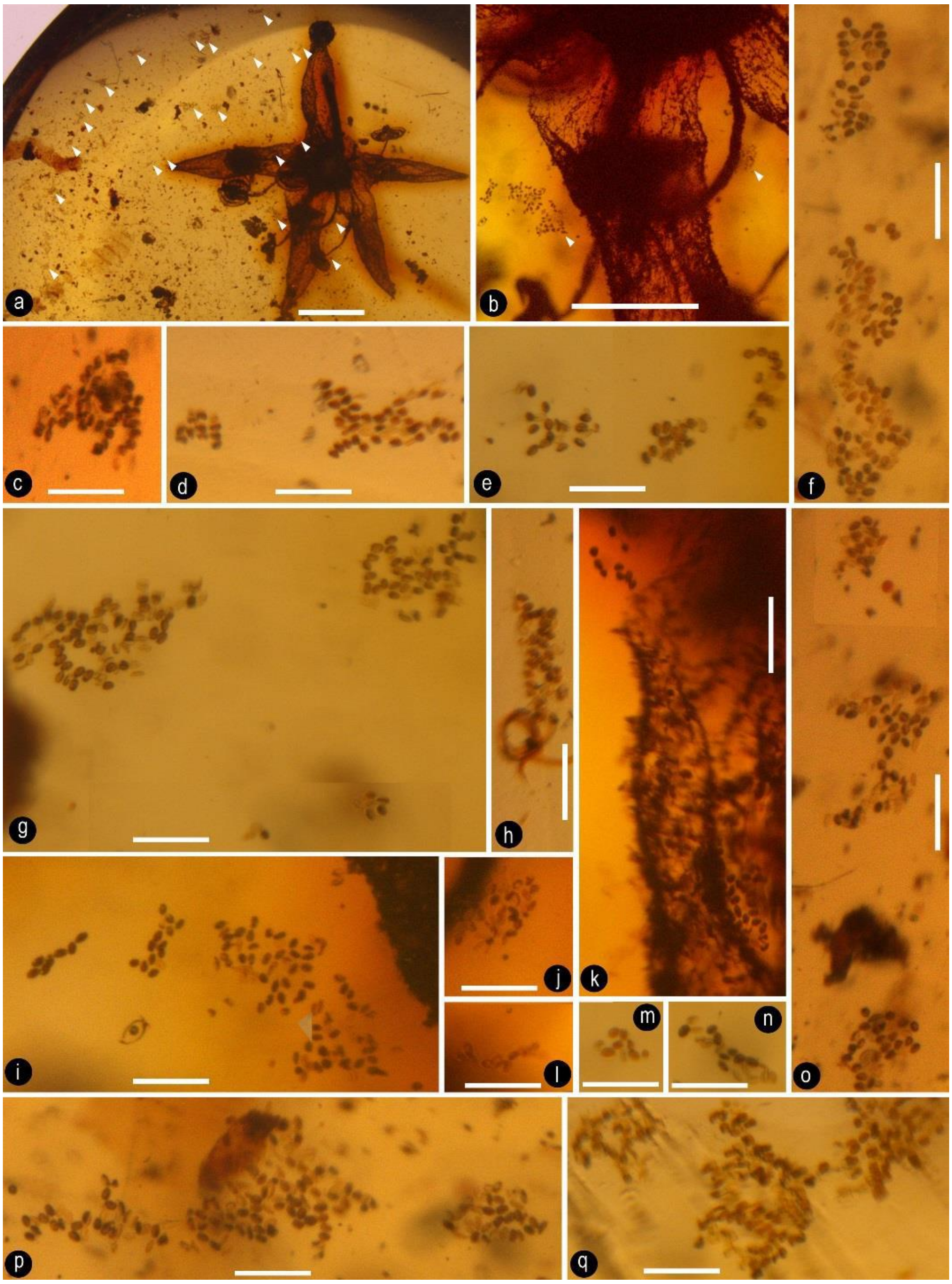

Supplementary Figure 1 Lijinganthus revoluta gen et sp. nov. and closely associated pollen grains in a Myanmar amber. All scale bars are 0.1 mm long unless otherwise annotated. A. The flower with pollen grain clumps (triangles) scattered around. Scale bar = 1 mm. B. Detailed view of a portion of the flower showing two pollen grain clumps (triangles) closely associated with the flower. Scale bar = 0.5 mm. C. 49 pollen grains in a clump. D. 50 pollen grains in two clumps. E. 39 pollen grains in two clumps. F. 111 pollen grains in two clumps. G. 112 pollen grains in four clumps. H. 23 pollen grains in a clump. I. 85 pollen grains in several clumps. J. 24 pollen grains in a clump. K. 29 pollen grains in two clumps. L. 8 pollen grains in a clump. M. 9 pollen grains in a clump. N. 18 pollen grains in a clump. O. 68 pollen grains in three clumps. P. 103 pollen grains in several clumps. Q. 84 pollen grains in three clumps.
